# Linking mobile money networks to “e-ROSCAs”: An experimental study

**DOI:** 10.1126/sciadv.abc5831

**Published:** 2021-01-01

**Authors:** Patrick Francois, Munir Squires

**Affiliations:** 1 Vancouver School of Economics, University of British Columbia, Vancouver, BC, Canada.; 2 Canadian Institute for Advanced Research, Toronto, ON, Canada.

## Abstract

We present results from a study in the Democratic Republic of the Congo that uses mobile money networks to run rotating savings and credit associations (ROSCAs), peer-to-peer finance groups ubiquitous across the developing world. We find high rates of contribution and ROSCA success. The unexpected success of such e-ROSCAs and their potential to extend banking to the bankless poor necessitate further exploration.

## Teaser

Peer-to-peer banking groups in the DRC show high rates of contribution, suggesting a way to reach the unbanked globally.

## INTRODUCTION

Most of the world’s poor have limited access to formal banking. The closest they come to borrowing in an institutionalized setting is via rotating savings and credit associations (ROSCAs), which are ubiquitous across the developing world (*1*–*4*).

ROSCAs meet regularly, require from each member a contribution amount at the meeting, and disperse summed contributions (the pot) to a single member. At the next meeting, which all must attend, members who have received the pot are excluded from receiving again but are required to contribute. This continues until all members have had a turn to receive the pot. ROSCAs are successful, or so it is believed, because of regular monitoring and selection among their members and the exertion of social pressure to ensure compliance (*5*).

Mobile money networks have become widespread in the developing world. However, these primarily facilitate transactions, not banking. Although borrowing is rarely available through these networks, they do raise an intriguing possibility. They make feasible “e-ROSCAs”: virtual ROSCAs implemented through linked mobile money accounts. Since both ROSCAs and mobile money are so widespread, if e-ROSCAs were to become feasible, then they would markedly extend borrowing and savings possibilities to many of the world’s bankless poor.

The primary impediment would seem to be that individuals who are connected only virtually would lack the monitoring, selection, and social pressure thought necessary to limit free-riding. High rates of abstention after receiving the pot would lead e-ROSCAs to unravel. While we know of one mobile e-ROSCA app (“Akiba”), it focuses on connecting individuals with preexisting offline relationships.

In the first field experiment of its kind, we set up and ran e-ROSCAs conducted via text message (SMS). We designed these e-ROSCAs so as to strip them of the peer monitoring present in traditional face-to-face ROSCAs. Participants were grouped anonymously and almost entirely unincentivized to contribute once they had received their allocation. Unexpectedly, participants chose to contribute in around 90% of cases, and almost two-thirds of participants reached the end of their cycle with full compliance. These results are not out of line with studies of African ROSCAs (*6*). However, existing studies are of face-to-face ROSCAs among repeat players known to each other, and we know of none that study “virtual” ROSCAs.

We augmented the fully anonymous baseline setting by adding lightweight analogs of features found in face-to-face ROSCAs. These elements are assortative sorting, information about group composition, and their interaction. We compute the contributions of these elements to e-ROSCA function and suggest how they can be used in the future design of such e-ROSCAs. These experiments provide encouraging evidence that mobile money networks could indeed undergird e-ROSCAs, opening up potentially enormous increases in access to finance for the very poor throughout the developing world.

### e-ROSCAs

**Fig. 1:**
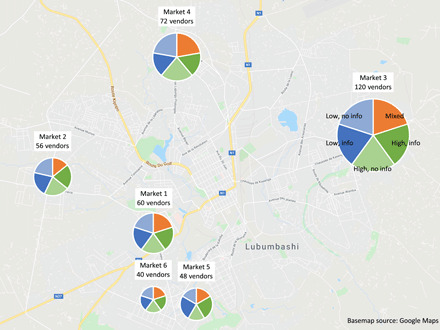
Market locations.

We set up and ran real-world e-ROSCAs in the Democratic Republic of the Congo (DRC) in June 2018. The experiment was run with shopkeepers from six markets in the city of Lubumbashi, as shown in Fig. 1. Recruitment methods and inclusion criteria are in the Supplementary Materials, along with an overview of our survey. Table S1 presents descriptive statistics of our sample of participating shopkeepers, as well as the broader sample from which they were drawn. In total, the e-ROSCA experiment was done with 396 participants, who were assigned to 99 four-person groups.

The e-ROSCA groups each consisted of four members and, to mimic the face-to-face ROSCAs that these shopkeepers use, had a daily contribution frequency. Before the first day, each player was told which of the 4 days would be their turn to receive the pot. Daily contributions from each player were 500 Congolese francs (CDF), and hence, the person assigned the pot received a maximum of 2000 CDF on their assigned day. To benchmark magnitudes, a portion of cooked maize “fufu,” the staple meal, costs 200 CDF. Participants were allocated 2000 CDF at the formation of their e-ROSCA and updated daily on their balance. More details on this endowment and other features of the design are discussed in more detail in Materials and Methods.

Players in the e-ROSCAs had no way of communicating with or identifying each other. We updated each player daily regarding how many players remained in their group (i.e., how many had, so far, made all of their contributions).

All participant interactions were directly with the research team, through SMS messages. Given potentially large enumerator effects (*7*), the strict control that SMS allows is an important feature of our study design. Examples of text messages received are given in the Supplementary Materials.

Players were reminded each morning, and throughout the day, that they could either pay their contribution or discontinue participation in the e-ROSCA. Decisions were made by replying to our SMS. A final message in the evening reminded players that not responding would be treated as nonpayment and result in their removal. Alternative treatments are discussed and quantified in the Supplementary Materials.

Since the e-ROSCAs would last only one cycle, a 1000-CDF “continuation value” was paid to each group member if and only if all of them were in good standing after day 4. This additional payment was meant to substitute for the value that participants receive from successfully concluding a ROSCA cycle and thereby being able to continue into future rounds. Traditional ROSCAs typically persist cycle after cycle until the group dissolves. As we will see, this payment still implied that most contribution decisions were strictly payoff reducing (SPR) after receiving the pot, a factor that we discuss at length.

We ran three types of e-ROSCA. A baseline (described up to now) without sorting and without information, a second set in which individuals were sorted on grounds likely to be correlated with e-ROSCA performance but not told of this, and a third set in which individuals were informed of that sorting.

In the sorting intervention, individuals were grouped into e-ROSCAs on the basis of their performance in a dictator game (DG; described below). In the information set, we also provided members with a coarse signal regarding this grouping.

### DG selection

Each participant played a DG, which was conducted before being assigned to an e-ROSCA. We told participants they would be paired with another participant from their market and could decide how to split 1000 CDF between him/herself and the other player. Both player and partner knew that they would remain anonymous, to eliminate any potential social consequences. Participants privately made their decision, by SMS, to reduce social desirability bias vis-a-vis the enumerator.

The mean proportion shared by players in the DG was 32%. Figure S2 shows the distribution of these choices. Those below the median for their market shared an average of 12%, and those above the median shared an average of 50%.

Nineteen of the 99 e-ROSCA groups (denoted as “Mixed”) were formed of a mix of players who were told nothing about each other. The other 80 groups (denoted as “Sorted”) were equally split into “high” and “low” DG generosity groups. Median DG players were randomly allocated to either high or low groups in such a way as to ensure equal numbers of each type. Players were not informed beforehand that some groups would be sorted, nor did they know that their choices in the DG would have any bearing on their group.

Half of each type of the Sorted groups were also assigned to an Information treatment. If so, then we informed them that their fellow group members were all either of high or low generosity. The wording of these messages is in the Supplementary Materials.

Figure S4 summarizes how participants were divided into these various groups. Within their groups, participants were randomly sorted from rank 1 to rank 4, determining which day they would receive their group’s pot.

### Payoff reducing contributions

In all ROSCAS, decisions on different days, for players of different ranks, are made with different incentives and carry some risk. This is easiest to see for rank 4 players. If they were to pay 500 CDF the first 3 days but receive no contributions on day 4, then they would lose 1500 CDF. If maximizing their own payoff, choosing to contribute in the first 3 days would involve an assessment of the trustworthiness of one’s group members.

However, participants may contribute even when it is clearly not in their material interest. For e-ROSCAs like the one that we designed to succeed, some players must be willing to contribute even when they know contributing reduces their net payoff. For example, a payment on day 2 is SPR for a player of rank 1. It entails a loss of at least 500 CDF because from day 2 onward, these players only make contributions and never receive. The potential viability of e-ROSCAs may depend crucially on these SPR decisions, which we study explicitly below.

## RESULTS

Participants paid their contributions 88% of the time, and a full 62% of participants paid in all four periods. Contributions are equally high when payoff reducing (89%). However, controlling flexibly for the day and a player’s rank does suggest that SPR contributions may be slightly less likely (−3.6 percentage points, *P* = 0.1). These results and the ones that follow in this section are reported in tables S3 and S4.

**Fig. 2:**
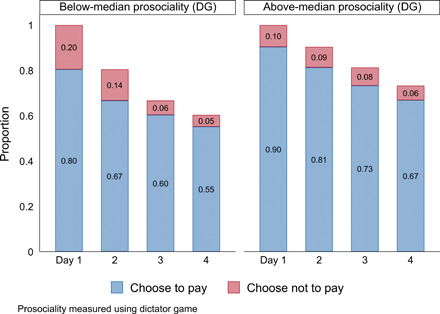
Above/below-median prosociality, days 1 to 4.

Our measure of prosociality strongly predicts e-ROSCA behavior: A 10–percentage point increase in DG sharing is linked to a 3–percentage point higher likelihood of making all four payments (*P* < 0.01). Above-median prosociality players contributed 90% of the time. Those below the median contributed 85% of the time and are 8 percentage points less likely to contribute when SPR (*P* < 0.1). For above-median players, the difference is only 2 percentage points (*P* > 0.1). The probability of making all four contributions was 55% for players below the median and 67% for those above it, as shown in Fig. 2. Some predictive power of sorting by DG is consistent with (*8*), which found behavioral prosociality to be stable across diverse games played over a period of several months.

**Fig. 3:**
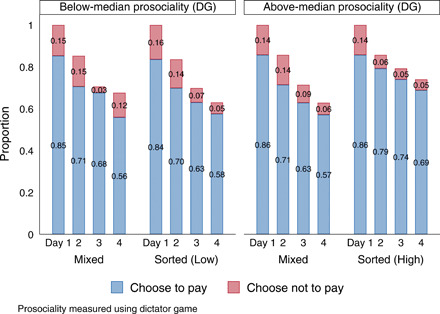
Effect of being sorted by type, days 1 to 4.

Sorting by prosociality (see Fig. 3) only seemed to matter for high-prosociality players. In total, 69% of above-median players in Sorted groups made all four contributions versus 57% in Mixed groups (*P* > 0.1). Among those below the median, 56% in Mixed groups made all four contributions versus 58% if sorted.

### The effect of information

**Fig. 4:**
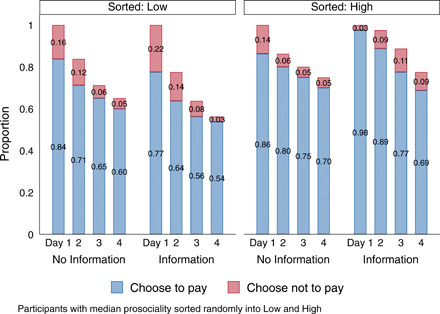
Effect of information, days 1 to 4.

Telling players that their group was all high-DG generosity had little effect on their likelihood of making all four contributions (70% versus 69%; see Fig. 4). However, this information increased the probability of a contribution on day 1—when players have no other information on their partners—from 86 to 98% (*P* < 0.01). For low types, knowing one’s group members were all low-type reduced players’ probability of making all four contributions from 60 to 54% (*P* > 0.1). First period contributions fell from 84 to 78% (*P* > 0.1).

The high-type signal seems to matter more (upward) in the first period than does the low-type signal (downward). However, the overall zero effect of information treatment on the high-type groups suggests that performance outweighs the type signal in later rounds.

In contrast, the low-type signal’s effects seem to persist. This may be because ROSCAs feature an increasing proportion of SPR decisions as the cycle progresses (in this case, 25% on day 2; up to 50% on day 3; and up to 75% on day 4). To low-prosociality players, who seem more sensitive to payoff consequences, sorting information may become gradually more salient because of the increasing frequency of SPR decisions. For high types, who do not appreciably alter their behavior for SPR decisions, it appears that the importance of sorting information relative to observed behavior progressively declines. Figure 4 shows the progression, day by day, of the number of players still contributing to their group for each of the 4 days, illustrating these results.

## DISCUSSION

Our priors were pessimistic about the baseline e-ROSCAs working. However, these anonymous, virtual, and weakly incentivized groups featured correct contributions 88% of the time, with 62% of players making all their payments. Even when making a contribution could only reduce one’s payoff, individuals contributed 90% of the time. A simple measure of prosociality was found to predict compliance and ROSCA success: A 10–percentage point increase in DG sharing predicted a 3–percentage point higher likelihood of a player, making all four payments.

These results seem to suggest that mobile money organizations could play a substantial banking-type role through the setting up of e-ROSCAs among their participants. We believe that this is worth exploring further. To quantify and benchmark potential efficiency gains, a Kenyan service called M-Shwari, partnered with a domestic bank, offers 30-day loans at 7.5% interest (138% annual equivalent rate) over a mobile money network. Our e-ROSCAs generate loans at zero interest.

Quantifying the welfare effects of e-ROSCAs is not yet possible without further evidence. However, a cost analysis in the Supplementary Materials suggests that they may offer substantial cost savings relative to traditional ROSCAs. Furthermore, evidence from the creation of savings groups across Africa suggests that, even in the presence of informal savings and credit, there may be large returns to providing more structured versions of these financial services (*9*, *10*).

Our e-ROSCAs have not used information on identity, repeat interaction, and location that mobile money networks would have available, yet members paid up in 90% of cases where direct payoff incentives dictated doing otherwise. Compliance could only be improved with increased incentivization. How much so, and in what contexts, deserves further scrutiny.

Using minimal indirect information about prosociality to sort members led to increases in e-ROSCA performance. This was enhanced by providing a coarse statement of that information to participants. Real-world mobile money networks would have greater opportunity to observe performance through time and could provide more incentivized sorting and finer information. The effects of these factors on e-ROSCA performance remain to be explored.

A limitation of traditional ROSCAs is their need for proximity. People must live close to each other to attend meetings, aggregate contributions, and disburse the pot. Since e-ROSCAs show high rates of compliance without using proximity, mobile networks could instead sort individuals from their pools of users, on the basis of magnitude or frequency of desired payments. How far ROSCA services could be improved via individually tailored e-ROSCAs also deserves exploration.

## MATERIALS AND METHODS

### Location and overview

Recruitment for this study was conducted at six markets in the city of Lubumbashi in the DRC. See Fig. 1 for a map of market locations, as well as the relative number of participants in each.

These open-air markets mostly consist of rows of small stalls where goods are sold during the day and taken away at night. The markets were chosen so as to be not only sufficiently large but also far enough from each other that information would be unlikely to spread across markets. This is relevant because the experiment was conducted sequentially across markets rather than simultaneously.

The study was conducted in three steps. First, a listing exercise was conducted to determine eligibility for the study from the general population of market vendors in a given market. Second, a more detailed survey was carried out with eligible market vendors to elicit various characteristics and play a DG by SMS. Last, the e-ROSCA was carried out remotely through SMS with a subset of participants who completed the survey. For each participant, the study lasted under 2 weeks and did not interfere with regular work activities.

### Sample selection

Participants in each market were recruited in the first place through a listing exercise, which was conducted with all willing shopkeepers. This listing exercise included only basic questions that would help determine eligibility for this study. The criteria for eligibility were the following: willing to participate in the study; 18 years of age or above; working from the same location for the following week; owns a phone that is exclusively theirs; uses their phone semiregularly and is able to send and receive SMS messages (basic digital literacy); willing to give us their phone number; knows how to send SMS without assistance; can read rudimentary French.

Some basic demographics (age, sex, and education) were also asked as part of the listing exercise, to allow for comparability with our final e-ROSCA sample. Descriptive statistics of participants in this phase of the study, and the subsequent phases, is in table S1.

Response rates in the listing exercise were very high. In the first four markets, there were 24 explicit refusals out of 1141 respondents, slightly over 2%. This figure may somewhat underestimate refusals if enumerators skipped vendors who were too busy to speak, for example, and omitted to return later. To minimize this problem, the listing protocol only required that the enumerator record the sex and approximate age of the respondent if they refused to participate. In the last two markets, some enumerators preempted the eligibility criteria by asking about phone ownership before starting the official listing exercise to save time. We have no reason to believe that the refusal rate would have been any higher in these last two markets.

Each market’s listing exercise lasted only 1 day. After the listing exercise, a list of selected survey participants was drawn up and assigned to enumerators for follow-up.

Selected participants were revisited in person by a member of the survey team and asked whether they were willing to continue participating in the study and complete the survey. Surveys were conducted one on one, and responses were entered in a tablet using SurveyCTO software.

After all surveys were completed in one market, respondents were invited to participate in the ROSCA portion of the study. The nature of the e-ROSCA and how it would function were explained to them in person during the survey exercise. Once invited, participants had to choose whether to join an e-ROSCA by sending an SMS reply. This provided an additional screen to limit the number of ROSCA participants who either had difficulty replying to SMS messages or did not have regular access to their phone.

Approximately 55% of the eligible market vendors decided to participate in an e-ROSCA, which we feel is encouragingly high. For a within-sample benchmark, 67% of the participants in our study have, at some point, been members of a ROSCA (table S1). This is consistent with high rates of participation in ROSCAs in many populations. For example, Anderson and Baland (*11*) point out that “Average membership [in ROSCAs] among adults ranges between 50 and 95 percent in the Republic of Congo, Cameroon, Gambia, and villages of Liberia, Ivory Coast, Togo, and Nigeria.” In addition, Afzal *et al*. (*12*) offer a ROSCA-like product with daily contributions to rural Pakistani women and find a 65% take-up rate. It is a common finding in this literature that there is substantial unmet need for suitable financial products (*13*).

### Survey questionnaire

For each participant, the survey exercise took up to an hour, stopping when the vendor received customers. Whereas the listing exercise lasted only a day in any given market, surveying all eligible participants took up to 5 days per market. The survey consisted of three key elements. The first was a questionnaire meant to gather key demographic variables and learn about their experience with (traditional) ROSCAs. The second was a DG, and the third was a description of the e-ROSCA and instructions on how it would function, followed by asking whether they would be interested in joining one.

The questionnaire included the following questions: religion; marital status; years worked in the market; daily earnings; number of shopkeepers in this market regularly interact with; current membership in a ROSCA; if member of a ROSCA and frequency of contributions (e.g., daily, weekly, and monthly).

Table S1 describes key variables from the three relevant samples. The listing sample includes all shopkeepers who agreed to participate in the listing exercise. We have relatively sparse data on this group, as we asked only a few questions at this stage. The survey sample consists of those who were selected to participate in the more detailed survey, who could be located, and agreed to participate. The ROSCA participants are a subset of the survey sample who expressed interest in joining an e-ROSCA and accepted their invitation by SMS within the allotted time.

Table S1A shows that those who participated in the survey are not randomly selected from the listing sample. They are more male, younger, more educated, and more likely to own a phone and to frequently use SMS. These differences stem mostly from our selection criteria—we required survey participants to own a phone and frequently use SMS. People who meet these criteria are more likely to be male, young, and educated. From within the survey sample, however, ROSCA participants are reasonably representative. Age and sex differences between these samples are not significant. ROSCA participants are more educated but only by an average of 0.17 additional years.

We can further compare the survey sample with ROSCA participants using survey responses, as in table S1B. Here, we see that ROSCA participants are representative of our survey sample along most measures. The only significant difference is in the share who are members of a daily (traditional) ROSCA: 23% of ROSCA participants versus 20% of the survey sample.

### Implementation of DG

The DG is a laboratory game generally used to determine levels of generosity or prosociality in an incentivized setting. The game consists of two players although only one player has an active role. The first player is allocated a certain amount of money and is asked how much of that money they want to give to the second player. The second player simply receives the money given to them by the first player. In our setting, the entire game was conducted through SMS messages between the participant and automated messages from a server later seen by the researchers. Conducting this exercise through SMS messages minimizes social desirability bias vis-a-vis the enumerator.

To begin the game, participants were required to send a message to the number provided to prompt the system. Then, an automated message was sent to the participant asking them how much they wanted to contribute. They could choose to give between 0 and 1000 CDF, in 100-CDF intervals. After sending their decision, they received a message explaining what their choice entailed for themselves and for their partner and gave them a chance to change their decision if so desired. A sample message exchange is shown in fig. S1.

### Other laboratory games

Immediately before the DG, participants made decisions related to risk aversion, which were not incentivized and intended primarily to familiarize participants with the process of decision-making through our SMS system. Following the DG, they were offered the option of paying to hide income from other shopkeepers in their market. These decisions are not studied in this paper.

### e-ROSCA design

e-ROSCA groups all consisted of four anonymous (to each other) participants. The e-ROSCAs all lasted 4 days, with each player expected to make a contribution to the pot on each of the 4 days and each receiving the pot on the day assigned to them.

One of the reasons that we chose to have daily contributions is that shopkeepers in this setting are most familiar with daily ROSCAs. Unlike ROSCAs with longer frequencies (e.g., weekly), they allow them to convert small daily contributions into larger sums. One of the challenges of managing a small business, rather than receiving a salary, is having to match irregular flows of revenue to lumpy wholesale purchases without spending down one’s capital (*14*).

Participants were allocated 2000 CDF at the formation of their e-ROSCA and updated daily on their balance and what effect contribution decisions would have on this balance. Each day’s contribution was 500 CDF, so in the worst-case scenario, they would end the game with no balance remaining and hence gain nothing from their participation in the e-ROSCA. In the best-case scenario, a player allocated to receive the pot on the first day would receive 1500 CDF (all four members contributing) and stop contributing in the second period, ending with a balance of 3500 CDF. Since other players can easily infer that the member of their group to first receive the pot would face this temptation, contributions to the group could easily unravel.

Providing participants with an endowment from which they could make their ROSCA contributions ensured that they were not at risk of losing their own money by participating. This feature of our design, however, raises a concern about external validity: Do the contribution rates that we measure depend on this initial transfer? There are two potential concerns related to the idea that participants were making decisions with “house money.” The first is that participants may have felt they could cheaply signal their “type” to the experimental team or to themselves by contributing more than they would have otherwise. The second concern is that participants may have felt less compunction “stealing the pot” (by exiting in the round after it was their turn to collect) since their group members were also using unearned income to make contributions.

Our experimental design does not directly speak to these motivations, since all participants received the same initial endowment. However, existing work using evidence from DG behavior finds that, in samples similar to ours, DG giving is not a function of whether the endowment to be transferred has been earned or not (*15*–*17*). This suggests that the effect of the initial endowment on contribution rates is likely to be small.

The remainder of this section details inclusion into the e-ROSCAs, the allocation to e-ROSCA groups, and the daily messages sent to e-ROSCA participants including the information treatments. Last, this section describes design features meant to limit SMS comprehension problems.

### Invitation to participate in e-ROSCAs

Survey respondents who expressed interest in participating in the e-ROSCA were invited to join by SMS, a few days after completing their survey. Figure S3 illustrates the e-ROSCA invitation message. A large fraction of invitees did not respond within the allocated time and therefore did not participate in an e-ROSCA. Participants were given a full day to respond to the invitation, and a few reminders were sent throughout the day.

### Procedure for allocation to groups

Participants were allocated to four-person e-ROSCA groups using the following procedure, which was done separately for each market. See fig. S4 for a visual demonstration as to how participants were allocated to groups. First, 80% of participants were randomly assigned to the Sorted treatment (stratified by being above, equal to, or below the median in the DG), while the remaining 20% of Mixed participants were matched to each other randomly and allocated to their four-person groups.

Second, individuals assigned to the Sorted treatment were classified as being of either High or Low prosociality. Anyone who gave more than the median amount in the DG was considered a High type, and anyone below the median was considered a Low type. Participants with DG giving exactly equal to the median for their market were randomly allocated to either High or Low type in such a way that both types were of equal size. All the Sorted participants were then assigned to their four-person group, consisting only of players of the same type as them (either High or Low).

Third, all Sorted groups were assigned to Information or No Information treatments with equal probability. Players in Information groups were told that their group consisted of only High (or Low) types. The remaining half, while being in a Sorted group, had no way of knowing this and were unaware that any other groups had been sorted.

Within each group, participants were randomly assigned an order in which they would be served. For example, if a participant was assigned day 2, then they would receive the pot on day 2. On the other days, they would contribute and another member would receive the pot.

Ideally, there would be a multiple of 20 participants in each market (five different types of group, four participants in each group). However, when this was not the case, additional groups were formed balancing across markets. See table S5 for a breakdown of allocation into groups across the six different markets. Table S2 shows that relevant characteristics are balanced across treatments, as expected given randomization.

### Starting information

The information given to participants at the beginning of the ROSCA is displayed in fig. S5. Participants were reminded that their group consists of four members and informed of the day on which they would be served. Furthermore, participants in the Information treatment group were sent a message indicating the generosity level of the rest of their group (fig. S5B). This message was either “The others in your group were generous in [the DG]. They shared a large portion of the 1000 CDF” or “The others in your group were not generous in [the DG]. They shared a small portion of the 1000 CDF.” Participants in the No Information treatment are sent a shorter placebo message (fig. S5A). The same number of messages is sent to each group so as not to provide differential interactions with participants. Throughout the e-ROSCA, this short informational sentence is the only difference in messages received between any of the groups.

### Daily contributions

Once the ROSCA began, participants received information on how many other group members contributed to the previous day. They also received a reminder of which day they would be served. With that information in mind, they were asked whether they would contribute to the daily pot. These messages were the same for every group, regardless of treatment. An example of what this set of messages looked like is shown in fig. S6. Figure S6A shows a player’s decision on day 1. Figure S6B shows a decision on day 3, having observed the actions of group members after the first 2 days. Here, the participant chose to contribute in the first 2 days, along with two of their three group members, and again chooses to contribute on day 3.

While players were informed each day about how many of their group members paid their contribution on the previous day, they were not told which players did or did not. That is, when told a player dropped out of their group, they did not know which round that player was meant to receive the pot.

One special case deserves mention. If a participant dropped out on the day it was their turn to receive the pot, or before it, then the other participants who contributed were refunded their contributions for that day. This is analogous to going to a meeting to make your payment where the person meant to receive the pot is absent—you keep your money. This is illustrated in fig. S7.

### Minimizing SMS comprehension problems

Since SMS-based decision-making in the e-ROSCA is unfamiliar to participants, we took the following steps to minimize comprehension problems. During the in-person survey, participants were asked to participate in SMS-based activities. The enumerator assigned to them would first explain the activity and the choice that the participant would be asked to make. Once the participant felt ready to make their decision, their enumerator told them to send a particular message to one of our “gateway” phones. This initial step guaranteed that participants owned a working phone able to send SMS messages. It also provided us with additional confirmation of a participant’s phone number. The gateway phones, once they received the initial prompt, would send an introductory message to the participant and then a message explaining what decision they had to make in the first game, also explaining how to submit their decision.

Participants made their choice by sending a reply to the gateway. Enumerators were coached to help with this process if needed but not to do it for them. After making each decision, they received a message confirming what they chose and giving them the option of amending their choice if desired. After proceeding through the three games, the enumerator recorded the ease with which the participant read the messages and sent their replies. This allowed us to screen for participants who had difficulty with these tasks. A few days after the survey, participants who signaled interest and who were selected to participate were sent an invitation message by SMS asking whether they would like to join a ROSCA. Having to send a valid reply to an SMS message without an enumerator present further screened people who did not typically keep their phone charged or with sufficient credit to send a reply.

While the system was automated to deal with large numbers of SMS conversations every day, it was adapted to capture the intent in a message rather than relying on a specific set of characters. For example, to make a daily contribution, the appropriate message was “YES.” However, if someone replied with obvious alternatives such as “yes,” “I contribute,” or “I do,” then they were considered to have said YES. Any messages that were not automatically recognized as a decision were reviewed by a member of the research team to decide how to treat the response. The manager of the survey team was also available to help participants throughout the duration of the study.

### End-of-game payouts

Payments to players were made once, after all experimental decisions were complete. For each market, once the ROSCAs ended (that is, after day 4), all players received a text confirming how much money they would receive, including payments from the DG. See fig. S8 for an example of messages sent at this stage. Note that each player was both a “sender” and a “receiver” in the DG and so could receive up to a maximum of 2000 CDF from this portion of the study. Players were matched with each other randomly and were not told, when making their DG decision, that they would also be a receiver. In addition to this, players received their balance from the ROSCA game, which could be between 500 and 3000 CDF. Last, one player per market was chosen at random to win an amount from the money-hiding game depending on their response. For a player who chose to hide the money that they won, they would get 5000 CDF, whereas the player who chose not to hide would get 10000 CDF and they were announced (by first name only) as the winner to other market vendors in the study through SMS.

As a general rule, payments were made through mobile money. Most of the market vendors already had a mobile money account. For those who did not, a network agent helped them open an account at their market.

## Supplementary Material

http://advances.sciencemag.org/cgi/content/full/7/1/eabc5831/DC1

Data and code for replication

Adobe PDF - abc5831_SM.pdf

Linking mobile money networks to “e-ROSCAs”: An experimental study

## SUPPLEMENTARY MATERIALS

Supplementary material for this article is available at http://advances.sciencemag.org/cgi/content/full/7/1/eabc5831/DC1

## References

[1] Bouman F. J. A., Indigenous savings and credit societies in the third world: A message. Sav. Dev. 1, 181–219 (1977).

[2] Besley T., Coate S., Loury G., The economics of rotating savings and credit associations. Am. Econ. Rev. 83, 792–810 (1993).

[3] S. Ardener, S. Burman, *Money-Go-Rounds: The Importance of Rotating Savings and Credit Associations for Women*, vol. 15 of *Cross-Cultural Perspectives on Women* (BERG, 1995).

[4] S. Rutherford, S. S. Arora, *The Poor and Their Money: Microfinance from a Twenty-first Century Consumer’s Perspective* (Practical Action Publishing, 2009).

[5] Anderson S., Baland J.-M., Moene K. O., Enforcement in informal saving groups. J. Dev. Econ. 90, 14–23 (2009).

[6] Gugerty M. K., You can’t save alone: Commitment in rotating savings and credit associations in Kenya. Econ. Dev. Cult. Change. 55, 251–282 (2007).

[7] Laajaj R., Macours K., Pinzon Hernandez D. A., Arias O., Gosling S. D., Potter J., Rubio-Codina M., Vakis R., Challenges to capture the big five personality traits in non-WEIRD populations. Sci. Adv. 5, eaaw5226 (2019). 31309152 10.1126/sciadv.aaw5226 PMC6620089

[8] Bolton G. E., Katok E., Zwick R., Dictator game giving: Rules of fairness versus acts of kindness. Internat. J. Game Theory. 27, 269–299 (1998).

[9] Karlan D., Savonitto B., Thuysbaert B., Udry C., Impact of savings groups on the lives of the poor. Proc. Natl. Acad. Sci. U.S.A. 114, 3079–3084 (2017). 28270615 10.1073/pnas.1611520114 PMC5373400

[10] Ksoll C., Lilleør H. B., Lønborg J. H., Rasmussen O. D., Impact of village savings and loan associations: Evidence from a cluster randomized trial. J. Dev. Econ. 120, 70–85 (2016).

[11] Anderson S., Baland J.-M., The Economics of roscas and intrahousehold resource allocation. Q. J. Econ. 117, 963–995 (2002).

[12] Afzal U., d’Adda G., Fafchamps M., Quinn S., Said F., Two sides of the same rupee? Comparing demand for microcredit and microsaving in a framed field experiment in rural Pakistan. Econ J. 128, 2161–2190 (2017).

[13] Karlan D., Ratan A. L., Zinman J., Savings by and for the Poor: A Research Review and Agenda. Rev. Income Wealth. 60, 36–78 (2014). 25792764 10.1111/roiw.12101 PMC4358152

[14] Karlan D., Mullainathan S., Roth B. N., Debt traps? Market vendors and moneylender debt in India and the Philippines. Am. Econ. Rev. Insights 1, 27–42 (2019).

[15] Cappelen A. W., Moene K. O., Sørensen E. Ø., Tungodden B., Needs versus entitlements—An international fairness experiment. J. Eur. Econ. Assoc. 11, 574–598 (2013).

[16] Barr A., Burns J., Miller L., Shaw I., Economic status and acknowledgement of earned entitlement. J. Econ. Behav. Organ. 118, 40–54 (2015).

[17] Jakiela P., How fair shares compare: Experimental evidence from two cultures. J. Econ. Behav. Organ. 118, 40–54 (2015).

[18] J. P. Henrich, R. Boyd, S. Bowles, E. Fehr, C. Camerer, H. Gintis, *Foundations of Human Sociality: Economic Experiments and Ethnographic Evidence from Fifteen Small-scale Societies* (Oxford Univ. Press, 2004).

[19] Yamagishi T., Mifune N., Li Y., Shinada M., Hashimoto H., Horita Y., Miura A., Inukai K., Tanida S., Kiyonari T., Takagishi H., Simunovic D., Is behavioral pro-sociality game-specific? Pro-social preference and expectations of pro-sociality. Organ. Behav. Hum. Decis. Process. 120, 260–271 (2013).

