# Supplementary material for: Linking mobile money networks to “e-ROSCAs”: An experimental study
Source: Sci Adv. 2021 Jan 1;7(1):eabc5831. doi: 10.1126/sciadv.abc5831 (PMC7775776; doi:10.1126/sciadv.abc5831)
Supplement: http://advances.sciencemag.org/cgi/content/full/7/1/eabc5831/DC1 [file supp_7_1_eabc5831__index.html]

Science Advances | Science AdvancesAAASSearchScience AdvancesMenu

## Supplementary Materials

# Linking mobile money networks to “e-ROSCAs”: An experimental study

Patrick Francois and Munir Squires

Download Supplement

**The PDF file includes:**

- Supplementary Text
- Tables S1 to S7
- Figs. S1 to S8
- References

**Other Supplementary Material for this manuscript includes the following:**

- Data and code for replication

**Files in this Data Supplement:**

- Adobe PDF - abc5831\_SM.pdf
- abc5831\_Data\_and\_code\_for\_replication.zip
