# Supplementary material for: Linking mobile money networks to “e-ROSCAs”: An experimental study
Source: Sci Adv. 2021 Jan 1;7(1):eabc5831. doi: 10.1126/sciadv.abc5831 (PMC7775776; doi:10.1126/sciadv.abc5831)
Supplement: http://advances.sciencemag.org/cgi/content/full/7/1/eabc5831/DC1 [file supp_7_1_eabc5831__1.pdf]

Science Advances
AAAS

advances.sciencemag.org/cgi/content/full/7/1/eabc5831/DC1

Supplementary Materials for

Linking mobile money networks to "e-ROSCAs": An experimental study

Patrick Francois and Munir Squires*

*Corresponding author. Email: munir.squires@ubc.ca

Published 1 January 2021, *Sci. Adv.* **7**, eabc5831 (2021)
DOI: 10.1126/sciadv.eabc5831

The PDF file includes:

Supplementary Text
Tables S1 to S7
Figs. S1 to S8
References

Other Supplementary Material for this manuscript includes the following:

(available at advances.sciencemag.org/cgi/content/full/7/1/eabc5831/DC1)

Data and code for replication

Supplementary Material

Supplementary Text

Bounding payment non-response

Participants knew that not replying would be considered a failure to pay their contribution. Since sending an SMS involves a small cost in terms of time and prepaid phone credits, someone choosing not to contribute may have opted to express their decision by not replying. We cannot clearly distinguish between a participant choosing this strategy from one who was too busy to answer, or whose phone was lost or not charged, but who did want to pay their contribution.

In traditional ROSCAs, not coming to a meeting to pay one's share of the pot is generally considered equivalent to defaulting. Making one's payment on time must be sufficiently important to each participant that they overcome whatever practical hurdles stand in the way of doing so on time. This is why, in implementing the game, we felt it was reasonable to treat "timed out" decisions as being equivalent to choosing not to contribute.

Though we have treated all individuals who miss a payment as doing so because they have decided not to contribute, in some cases they intended to make their contribution but for some reason did not reply by that day's deadline. Reasons people gave us are that they left their phones at home, they could not charge their phone, they ran out of SMS credits, or they were busy.

We tried to minimize these "accidental No's" by screening participants at multiple stages (listing, survey, invitation) on their stated and actual ability to respond to SMS messages. Moreover, during the survey and at the beginning of the e-ROSCA, participants were provided with a small amount of airtime which was sufficient to send multiple SMS messages. Lastly, while there is a small cost to sending each message, the amounts to gain or lose in the e-ROSCA are much larger.

Nonetheless, we can use reasonable assumptions to bound the fraction of decisions where players did not respond but meant to contribute. While 12% of decisions entailed non-payment, in only 2% of decisions did someone explicitly choose not to contribute. An upper bound would be to treat all such "time-out" decisions as Yes'es instead of No's, and therefore not pay that day's contribution in only 2% of cases. A lower bound could come from the cases where someone did not answer (and hence did not pay) on the day that they were scheduled to receive the pot. Choosing not to contribute on such a day is hard to rationalize for any reasonable set of preferences. We find that people whose turn it is to receive the pot fail to answer with a decision 6% of the time. Under the assumption that the proportion of individuals who miss payment under those circumstances is no smaller than the number of individuals who miss payment when they are paying but not receiving the pot, the 12% of decisions where

someone did not pay their contribution is reduced to 6%. Correcting for players who may have wished to contribute but missed one of their payments by mistake, the share of decisions to contribute out of all decisions made increases from 88% to a lower bound of 94%, and an upper bound of 98%.

Comparison of High and Low Dictator Game groups

The key dimension of heterogeneity in our sample is whether an individual chose to transfer a high or low amount to their partner when playing the Dictator Game. This has been shown in multiple studies to be an important predictor of prosocial behavior (*8*, *18*, *19*). Table S6 describes the differences between players with above and below median generosity (fraction transferred to their partner). First, notice that the above-median sample gave 50% of their allocation to their (anonymous) partner, while the below-median sample gave 12%. The group of people who gave exactly their market's median gave an average of 33%, but the number of people in this group is too small to be separately informative, so we focus on comparing above and below median participants.

While those with above and below median generosity are similar in age and education, women are less likely to be above median generosity than men. Daily earnings are one-third higher in the above-median generosity group, though the difference is not statistically significant.

Cost-Analysis

One of the most attractive features of ROSCAs is how inexpensive they are to operate. First and most importantly, they do not require external capital. Each period the members themselves provide the capital that becomes the pot. Because of this, and because the members themselves bear the cost of late repayment or default, the only costs associated with ROSCAs are (1) setting up groups, and (2) conducting the daily (or weekly, monthly, etc) sessions to exchange money. We provide some rough calculations of approximate costs of e-ROSCAs relative to the traditional sort for illustrative purposes.

The cost of setting up an e-ROSCA mostly involves the sign-up cost for a given individual. In our study, this meant paying an enumerator to visit each market vendor, collect some data from them, and explain how the e-ROSCA would operate. While enumerators spent an average of 45 minutes with each respondent during the survey, about 20 minutes of this was spent on survey modules and activities that were not related to the functions above. This translates to about $2 per participant. Note that this is a fixed cost per participant, which could be amortized over many rounds of e-ROSCAs. In principle this could be reduced if mobile money agents (who are widely available in these types of settings) marketed e-ROSCAs themselves, and were paid a fee for enrolling new members.

The costs of group creation for traditional (face-to-face) ROSCAs are less clear. The key challenge in these groups is matching people who have the same needs in terms of frequency

and size of contributions as well as group size, which determines the pot size and how often you receive it. It is not clear how to evaluate the implicit cost of this matching process. As a coarse lower bound on this cost, an hour of each participant's time seems reasonable for this process.

The cost of conducting the sessions of an e-ROSCA is low. These can be thought of as the cost of SMS messages, the mobile money transfer fees, and the opportunity cost of time used to make a decision and implement it. The cost per SMS message sent, averaged over the three mobile networks we used, is below 0.002 USD. If each session required five SMS messages (including reminders), this would equal 0.01 USD per session. Mobile money transfer fees are generally zero within a mobile money network. That is, sending mobile money to another user of your network is free for both sender and recipient. (In our case, we facilitated transfers across mobile operators, but in practice members of the same group would likely be required to be on the same mobile money platform.) Finally, the time needed to make a decision and implement it is likely no more than five minutes per session.

In a traditional ROSCA, by comparison, the time needed per session involves both travelling to the meeting place and conducting the exchange of money. While we do not have systematic data on this, thirty minutes is likely a conservative lower bound. Another element, which we do not attempt to quantify, is the risk of theft involved in having regular meetings where substantial sums of money are involved. Anecdotally, many ROSCA members are wary of disclosing where and when their meetings happen, and often alternate the location of meetings (for example cycling through the houses of the group's members) to avoid drawing attention.

An additional cost associated with an e-ROSCA is the hardware and software required. We used Telerivet, a web-based service, to manage the SMS-based communication platform. The plan we used costs $120 USD per month, and included substantial spare capacity (for example, up to 5000 messages per day, and up to 10,000 end users). We used three simple Android mobile phones to act as gateways, each of them about $60 USD. While these costs are surely concave in the number of e-ROSCA members, we assume they scale linearly for simplicity. Further, we assume that the capacity we used in our study would be sufficient for 1000 e-ROSCA members.

These costs are compiled and aggregated in table S7. In most cases this is simply a tabulation of the costs described above. Assuming linearity of all per-user costs in e-ROSCAS allows these not to vary with the number of people. Columns 1-3 show the effect of changes in the number of sessions (meetings) per ROSCA member, from 10 to 100. The number of sessions here is defined to include not just one ROSCA cycle but the entire lifespan of a person's participation, spanning potentially many cycles. As a benchmark, the median traditional ROSCA member in our sample had participated in approximately 90 sessions as of their survey date. Panels A and B suggest that while money costs are higher for e-ROSCAs, they potentially offer substantial time savings for their members.

Panel C summarizes the cost ratio of virtual and face-to-face ROSCAs. A key parameter necessary to do so is a measure of opportunity cost of time for ROSCA members. Specifically, the higher the value of time, the larger the cost savings of e-ROSCAs. Likewise, the more sessions an individual is expected to participate in, the larger the cost savings of e-ROSCAs. For most of the range of parameters we think are reasonable, e-ROSCAs offer a substantial cost advantage over traditional ROSCAs. However, we emphasize again that these back-of-the-envelope estimates should be taken as simply suggestive.

Deviations from Protocol

Deviations from study protocol are listed below.

1. In the last two markets, some enumerators pre-empted the eligibility criteria by asking shop-owners if they owned a phone (a more likely disqualifier) before starting the official listing exercise.
2. Unlike in markets 1-5, in the 6th market the listing and survey activities were done on the same day. This was done due to time considerations. For the first 5 markets, enumerators spent 4 or 5 consecutive days in the same market. This allowed the enumerators to spend a full day to do the listing exercise and then use the remaining days to follow-up with the questionnaire. Due to time constraints, participants that were deemed eligible (using rules coded into the data-entry software) proceeded immediately to the survey rather than being revisited.
3. Markets (including their listing, survey and e-ROSCA phases) were done sequentially. However, markets 4 and 5 were done simultaneously instead of sequentially. There was also a one-day overlap in markets 5 and 6.
4. Some vendors did not have a mobile money account and expressed no interest in opening one. These people were provided with cash instead for the end-of-game payout.
5. For most markets, reminders were only done through SMS messaging, however in one market, eligible vendors from the survey stage of the study were phoned to assess and garner interest. This was done in part to have some sense of why vendors would or would not choose to participate in the e-ROSCAs.
6. On the second day of e-ROSCAs in the third market, participants were asked to make their daily contributions prior to providing information on how many members had contributed the previous day. To address this, the missing information was sent and participants who had already decided whether to contribute were asked to confirm their choice. None of these members changed their decision given the new information.

Supplementary Tables

Table S1: Summary statistics

	Listing sample		Survey sample		ROSCA participants		Listing vs. ROSCA	Survey vs. ROSCA
	Mean	SD	Mean	SD	Mean	SD	*p*-value	*p*-value
Panel A. Initial listing variables								
Female	0.34	0.47	0.21	0.41	0.20	0.40	0.00	0.52
Age	32.41	10.32	30.23	8.68	30.03	8.39	0.00	0.49
Education (yrs)	11.35	1.70	11.80	1.28	11.97	1.15	0.00	0.00
Owns a mobile phone	0.89	0.31	1.00	0.00	1.00	0.00	0.00	–
Frequent use of SMS	0.82	0.38	0.99	0.08	0.99	0.09	0.00	0.82
Panel B. Survey variables								
Earnings per day (USD)	–	–	16.64	42.35	16.34	40.43	–	0.83
Dictator share given	–	–	0.32	0.21	0.32	0.21	–	0.76
ROSCA member	–	–	0.31	0.46	0.33	0.47	–	0.24
Daily ROSCA member	–	–	0.20	0.40	0.23	0.42	–	0.04
Ever ROSCA member	–	–	0.67	0.47	0.69	0.46	–	0.20
Observations	1,400		721		396			

Notes: The last two columns' p-values are derived from an F-test that mobile ROSCA participation does not predict the balance variable among the larger sample (the listing and survey samples, respectively). 'Dictator share given' is the proportion shared with an anonymous second player in a dictator game by SMS before the start of the mobile ROSCA experiment and is a proxy for generosity. 'ROSCA member' and 'Daily ROSCA member' are variables indicating whether a participant is a member of a (daily) ROSCA outside of the context of the mobile ROSCA experiment.

Table S2: Balance

	Sorting Treatment			Information Treatment		
	Mixed	Sorted	p-value	No info	Info	p-value
Female	0.19	0.21	0.74	0.22	0.19	0.58
	(0.05)	(0.02)		(0.03)	(0.03)	
Age	29.22	30.21	0.36	29.66	30.76	0.24
	(0.97)	(0.47)		(0.64)	(0.68)	
Education (yrs)	12.08	11.94	0.34	11.92	11.96	0.74
	(0.11)	(0.07)		(0.09)	(0.10)	
Owns a mobile phone	1.00	1.00		1.00	1.00	
	(0.00)	(0.00)		(0.00)	(0.00)	
Frequent use of SMS	1.00	0.99	0.40	0.99	0.99	0.56
	(0.99)	(0.01)		(0.01)	(0.01)	
Earnings per day	16.33	16.34	1.00	16.05	16.63	0.90
(USD)	(4.91)	(2.27)		(3.04)	(3.05)	
Dictator share given	0.33	0.31	0.64	0.31	0.31	1.00
	(0.03)	(0.01)		(0.02)	(0.02)	
ROSCA member	0.41	0.31	0.11	0.34	0.29	0.34
	(0.06)	(0.03)		(0.04)	(0.04)	
Daily ROSCA	0.29	0.22	0.17	0.21	0.22	0.89
member	(0.05)	(0.02)		(0.03)	(0.03)	
Observations	76	320		160	160	

Notes: The p-values are derived from a difference of means t-test. Standard errors are reported in parentheses.

Table S3: Payoff-reducing decisions, prosociality and participation

Panel A. Payoff-reducing decisions and participation.

	Dep var: Chooses to pay (binary)	
SPR	0.010	-0.036
	(0.019)	(0.022)
Day and rank controls	N	Y
Number of decisions	1,302	1,302

Panel B. Payoff-reducing decisions and participation by prosociality.

	Dep var: Chooses to pay (binary)	
SPR	-0.085	-0.018
	(0.045)	(0.026)
Prosociality	Below-median	Above-median
Day and rank controls	Y	Y
Number of decisions	535	645

Panel C. Prosociality and participation in all 4 days.

	Dep var: Makes all 4 payments (binary)
Dictator share	0.306
	(0.113)
Day and rank controls	N
Number of participants	395

Panel A reports the correlation of a decision being strictly payoff-reducing (SPR) with the binary decision to pay, first without controls, then after categorical controls for the day and the player's rank (order) in the e-ROSCA. Panel B reports the controlled correlation between SPR and participation for below-median and above-median prosociality players (according to dictator game shares), respectively. Panel C reports the uncontrolled correlation between players' dictator game shares and whether they make all 4 payments. Standard errors clustered by e-ROSCA group reported in parentheses.

Table S4: Contributions, prosociality, and treatment groups

Panel A. Share of contributions paid by prosociality

	Share paid overall	N	Share paid by prosociality			
			Low	High	Diff	SE
All contributions	0.88	1,302	0.85	0.90	0.05	0.02
Day 4 contributions	0.62	396	0.55	0.67	0.12	0.05

Panel B. Share of contributions paid by prosociality and sorting (No information treatment)

	Low prosociality				High prosociality			
	Mixed	Sorted	Diff	SE	Mixed	Sorted	Diff	SE
Day 4 contributions	0.56	0.58	0.02	0.10	0.57	0.69	-0.12	0.10

Panel C. Share of contributions paid by prosociality and information treatment (Sorted groups)

	Low prosociality				High prosociality			
	No info	Info	Diff	SE	No info	Info	Diff	SE
Day 4 contributions	0.60	0.54	-0.06	0.08	0.70	0.69	-0.01	0.07
Day 1 contributions	0.84	0.78	-0.06	0.06	0.86	0.97	0.11	0.04

Table S5: Allocation to treatment group, by market

Sorted						
		High Dictator Group		Low Dictator Group		
Market	Mixed	Information	No information	Information	No information	Total
1	12	12	12	12	12	60
2	8	12	12	12	12	56
3	24	24	24	24	24	120
4	16	12	16	12	16	72
5	8	12	8	12	8	48
6	8	8	8	8	8	40
Total	76	80	80	80	80	396

Notes: Each row gives the allocation of participants across control and treatment groups within each of the 6 markets where participants were recruited.

Table S6: Comparison by generosity in dictator game

	(1) Below Median	(2) Market Median	(3) Above Median	(4) (1) vs. (3), p-value
Female	0.24	0.23	0.16	0.05
	(0.03)	(0.07)	(0.03)	
Age	29.40	31.91	30.25	0.33
	(0.62)	(1.81)	(0.60)	
Education (yrs)	11.89	12.00	12.03	0.25
	(0.09)	(0.16)	(0.08)	
Owns a mobile phone	1.00	1.00	1.00	-
	(0.00)	(0.00)	(0.00)	
Frequent use of SMS	0.99	1.00	0.99	0.61
	(0.01)	(0.00)	(0.01)	
Earnings per day (USD)	13.11	8.22	20.72	0.10
	(2.58)	(1.94)	(3.68)	
Dictator share given	0.12	0.33	0.50	0.00
	(0.01)	(0.01)	(0.01)	
ROSCA member	0.34	0.37	0.32	0.63
	(0.04)	(0.08)	(0.03)	
Daily ROSCA member	0.23	0.26	0.22	0.90
	(0.03)	(0.07)	(0.03)	
Ever ROSCA member	0.69	0.71	0.68	0.83
	(0.04)	(0.08)	(0.03)	
Observations	174	35	187	

Notes: Columns (1)-(3) report means for mobile ROSCA participants whose dictator shares respectively below, equal to, or above the median dictator share chosen with their market. Standard errors for these means are reported in parentheses. Column (4) reports p-values derived from a difference means t-test comparing below- and above-median respondents.

Table S7: Cost of e-ROSCAs per person

			Sessions		
**Panel A: e-ROSCA**			10	50	100
Group formation cost	*Per person*				
1.1) Signup cost		$2	$2.00	$2.00	$2.00
Meeting costs	*Per session*				
2.1) SMS messages		$0.01	$0.10	$0.50	$1.00
2.2) Time cost (5 min)		.083 hrs	.8 hrs	4.2 hrs	8.3 hrs
Technology overhead costs	*Per 1000 ppl*				
3.1) Software service		$120	$0.12	$0.12	$0.12
3.2) Hardware (gateway phones)		$180	$0.18	$0.18	$0.18
	Total money cost (USD)		$2.40	$2.80	$3.30
	Total time cost		.8 hrs	4.2 hrs	8.3 hrs
**Panel B: Traditional ROSCA**					
Group formation cost	*Per person*				
1.1) Signup cost		1 hrs	1 hrs	1 hrs	1 hrs
Meeting costs	*Per session*				
2.1) Time cost (30 min)		.5 hrs	5 hrs	25 hrs	50 hrs
Technology overhead costs					
None					
	Total money cost (USD)		$0.00	$0.00	$0.00
	Total time cost		6 hrs	26 hrs	51 hrs
**Panel C: Cost ratio, e-ROSCA vs Traditional**					
		$0.25	1.74	0.59	0.42
Value of time (per hour)		$0.5	0.94	0.38	0.29
		$1	0.54	0.27	0.23

Supplementary Figures

Figure S1: Sample message exchange

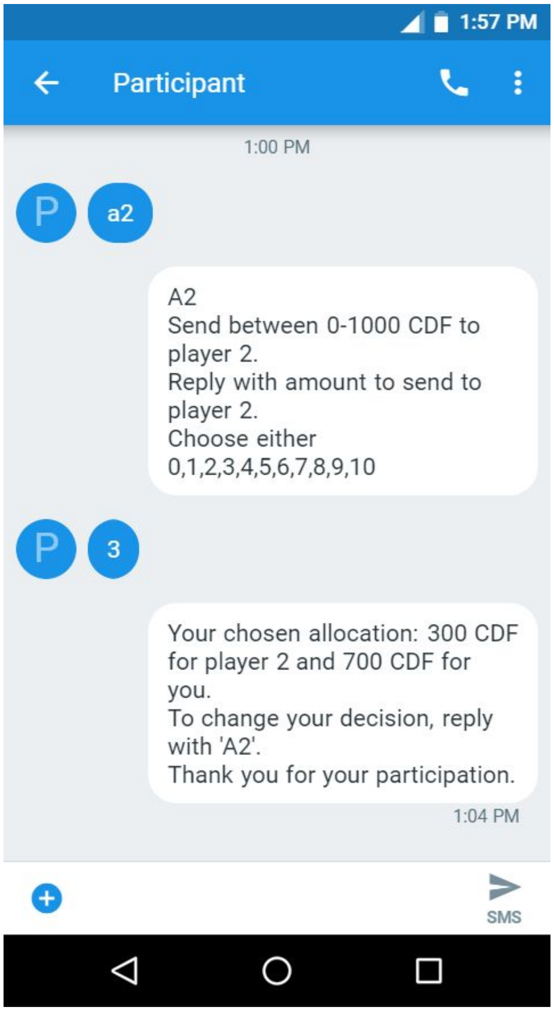

Figure S2: Distribution of choices in Dictator Game (DG)

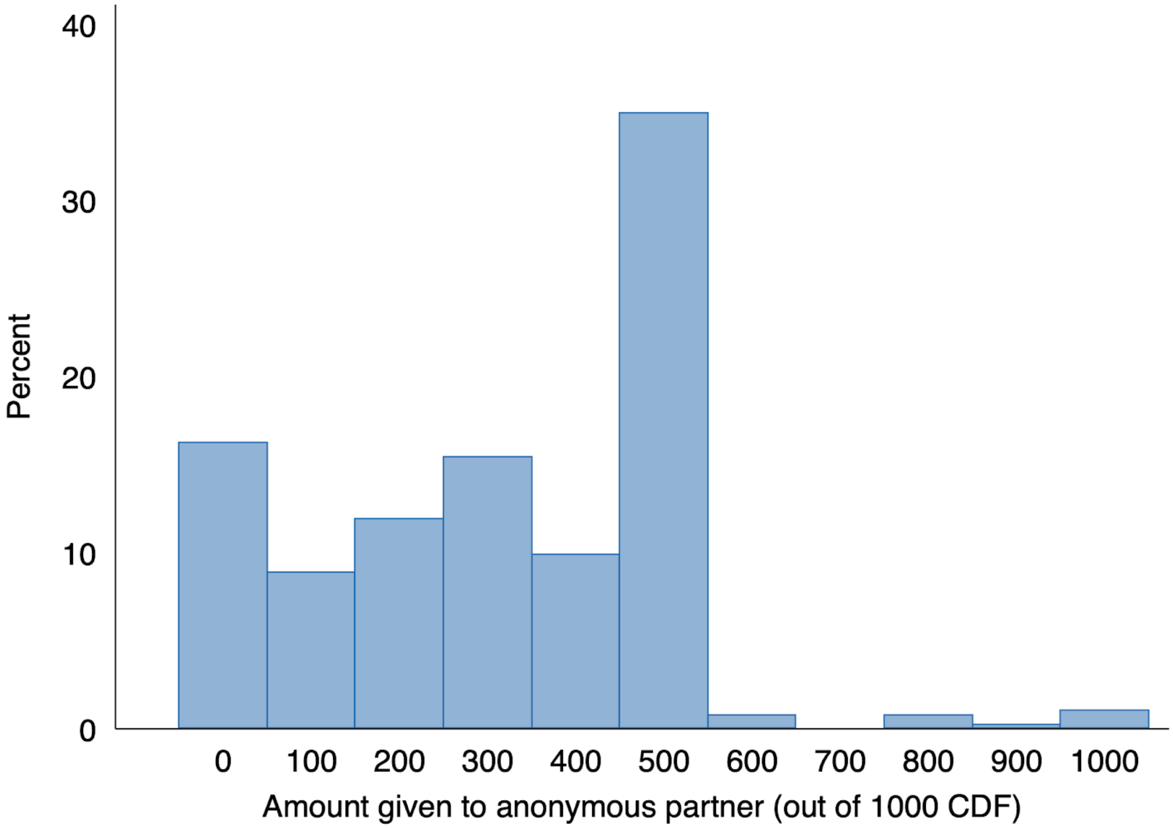

Figure S3: e-Rosca invitation message

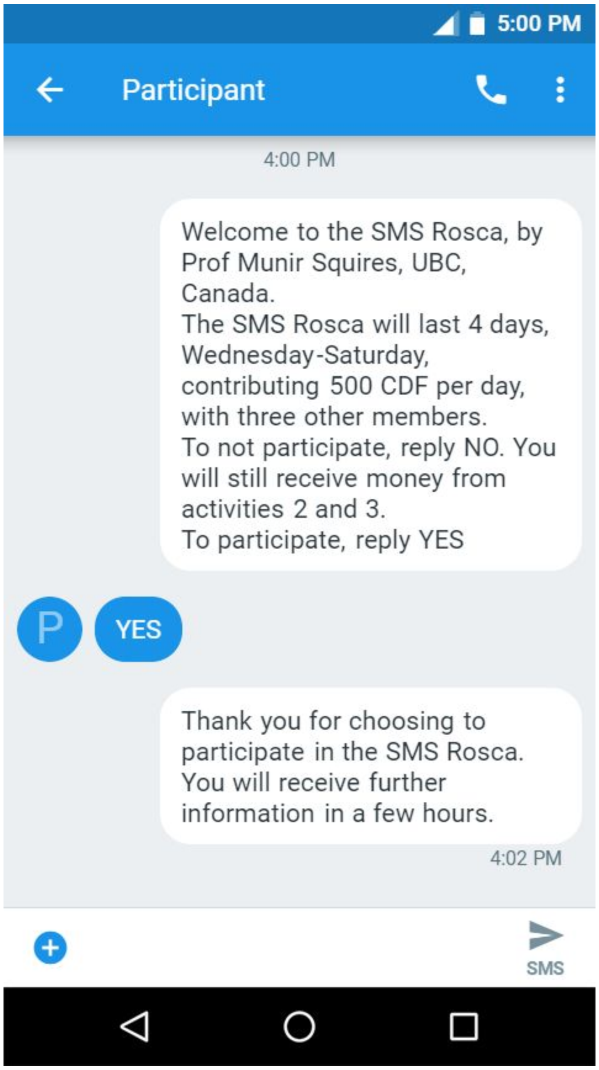

Figure S4: Study Design

Schematic of treatment assignment in a hypothetical market. The protocol presented here was followed in all markets, with the exact share of vendors assigned to each category varying slightly based on market size.

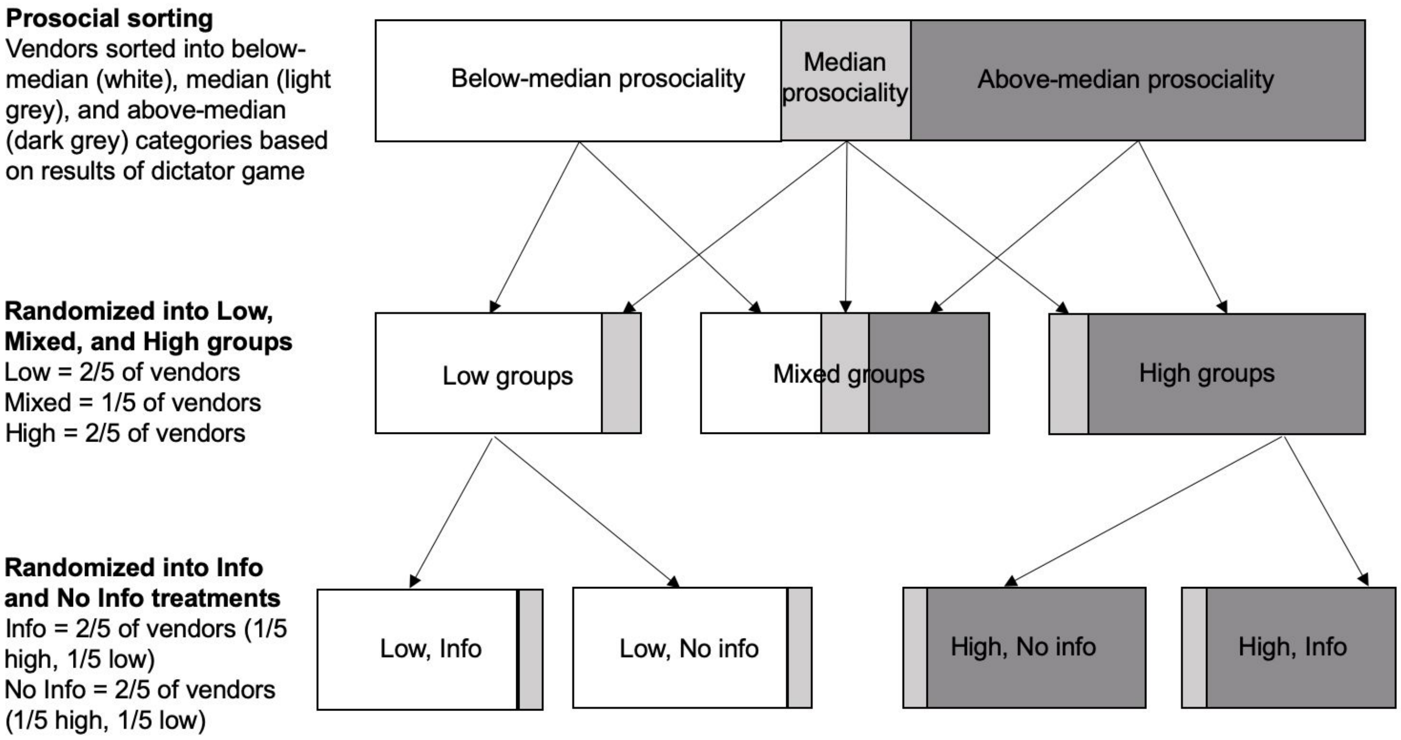

Figure S5: e-ROSCA setup messages

Panel A: No Information treatment

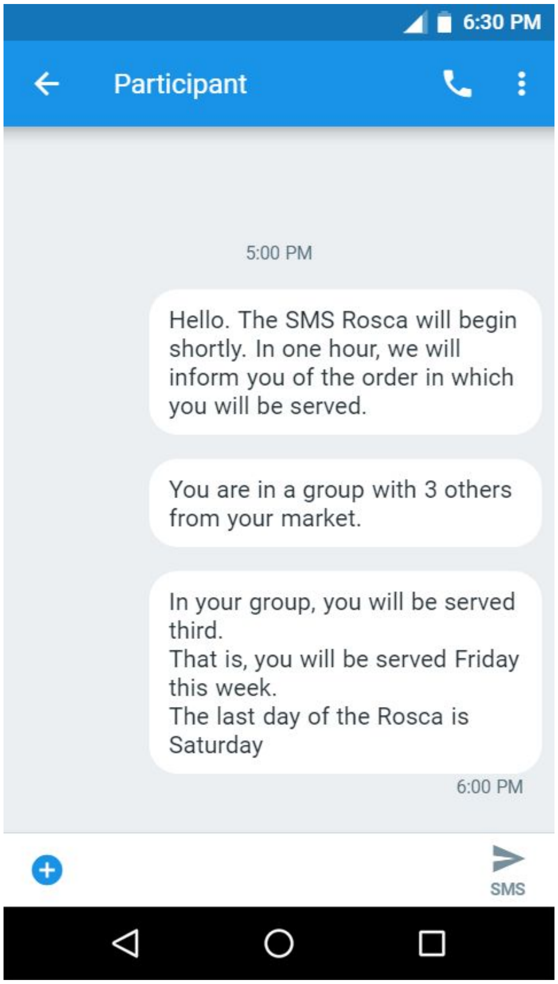

Panel B: Information treatment

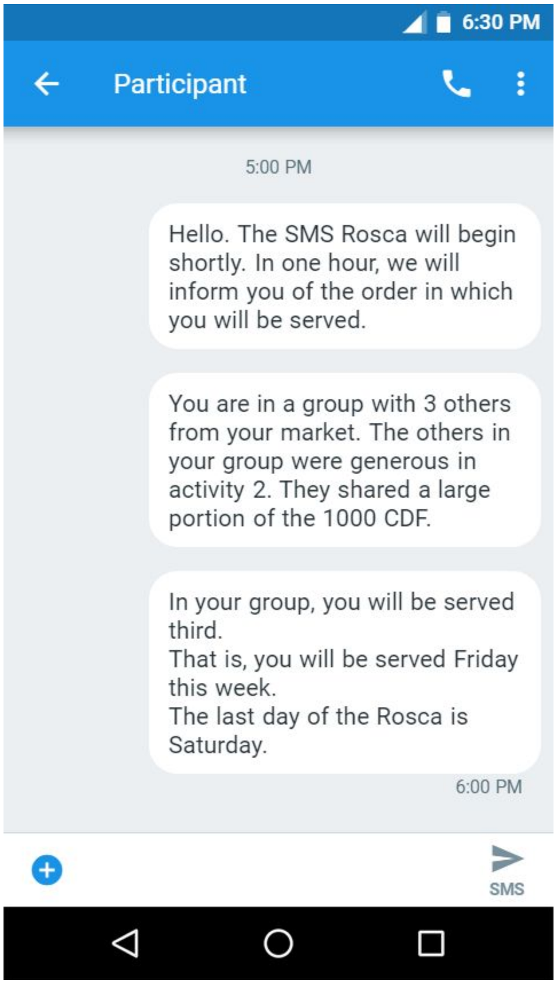

Figure S6: Daily contribution messages

Panel A: Day 1 of 4

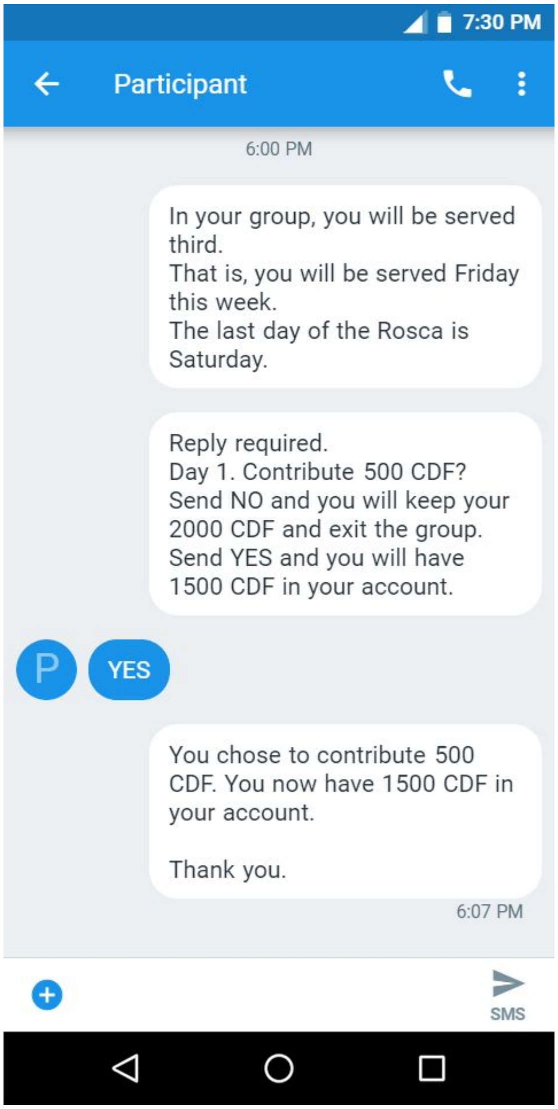

Panel B: Day 3 of 4

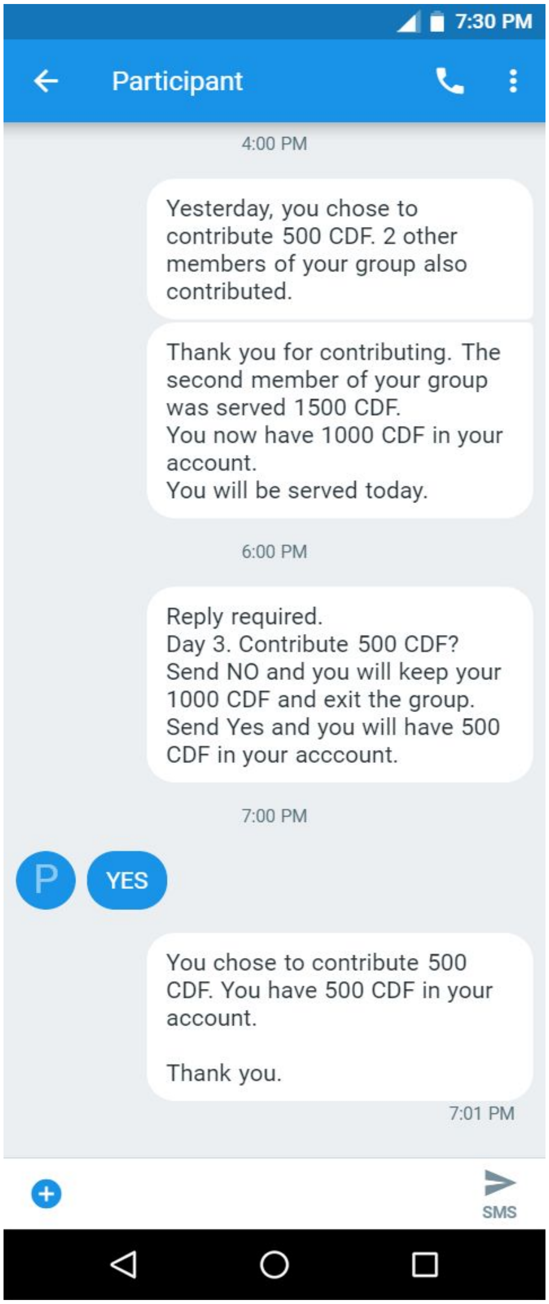

Figure S7: Contribution returned message

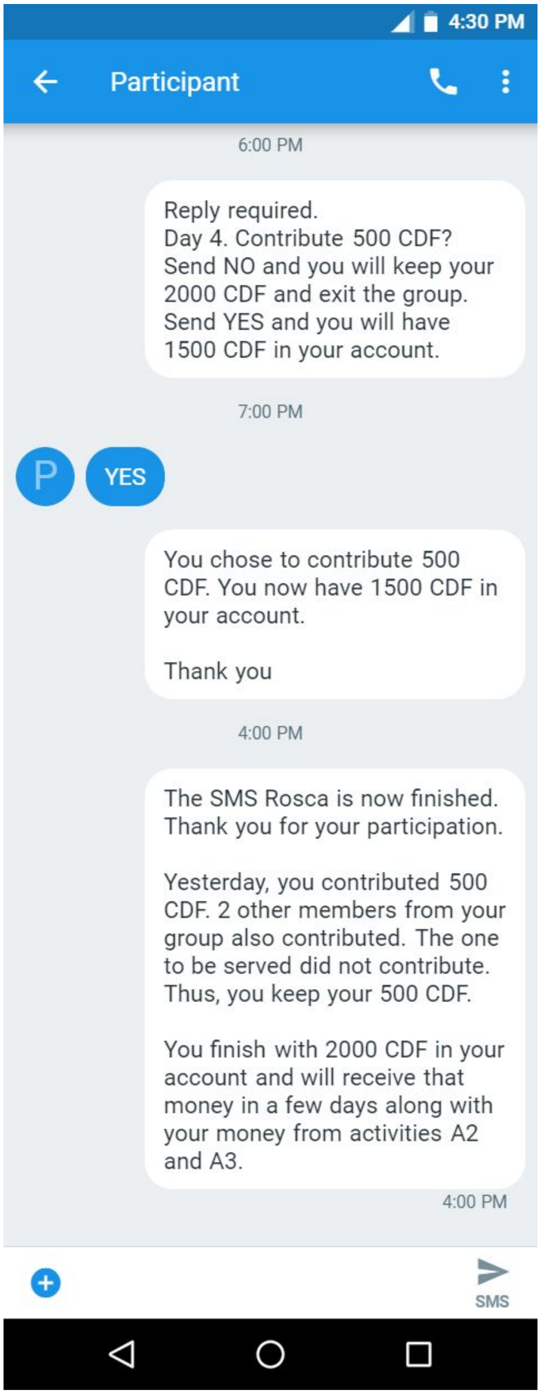

Figure S8: Payment message

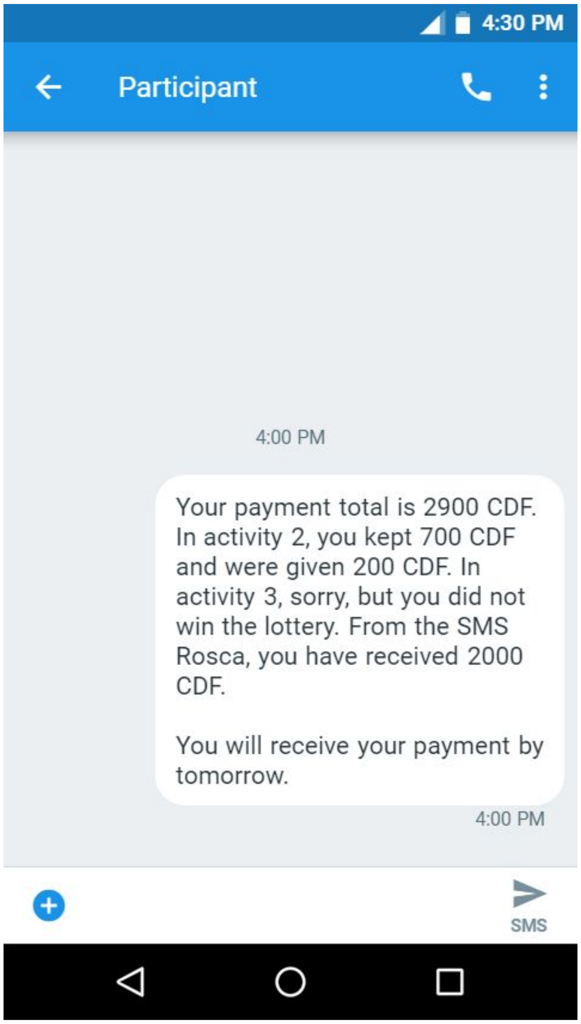

REFERENCES AND NOTES

1. F. J. A. Bouman, Indigenous savings and credit societies in the third world: A message. *Sav. Dev.* **1**, 181–219 (1977).
2. T. Besley, S. Coate, G. Loury, The economics of rotating savings and credit associations. *Am. Econ. Rev.* **83**, 792–810 (1993).
3. S. Ardener, S. Burman, *Money-Go-Rounds: The Importance of Rotating Savings and Credit Associations for Women*, vol. 15 of *Cross-Cultural Perspectives on Women* (BERG, 1995).
4. S. Rutherford, S. S. Arora, *The Poor and Their Money: Microfinance from a Twenty-first Century Consumer's Perspective* (Practical Action Publishing, 2009).
5. S. Anderson, J.-M. Baland, K. O. Moene, Enforcement in informal saving groups. *J. Dev. Econ.* **90**, 14–23 (2009).
6. M. K. Gugerty, You can't save alone: Commitment in rotating savings and credit associations in Kenya. *Econ. Dev. Cult. Change.* **55**, 251–282 (2007).
7. R. Laajaj, K. Macours, D. A. Pinzon Hernandez, O. Arias, S. D. Gosling, J. Potter, M. Rubio-Codina, R. Vakis, Challenges to capture the big five personality traits in non-WEIRD populations. *Sci. Adv.* **5**, eaaw5226 (2019).
8. G. E. Bolton, E. Katok, R. Zwick, Dictator game giving: Rules of fairness versus acts of kindness. *Internat. J. Game Theory.* **27**, 269–299 (1998).
9. D. Karlan, B. Savonitto, B. Thuysbaert, C. Udry, Impact of savings groups on the lives of the poor. *Proc. Natl. Acad. Sci. U.S.A.* **114**, 3079–3084 (2017).
10. C. Ksoll, H. B. Lilleør, J. H. Lønborg, O. D. Rasmussen, Impact of village savings and loan associations: Evidence from a cluster randomized trial. *J. Dev. Econ.* **120**, 70–85 (2016).
11. S. Anderson, J.-M. Baland, The Economics of roscas and intrahousehold resource allocation. *Q. J. Econ.* **117**, 963–995 (2002).
12. U. Afzal, G. d'Adda, M. Fafchamps, S. Quinn, F. Said, Two sides of the same rupee? Comparing demand for microcredit and microsaving in a framed field experiment in rural Pakistan. *Econ J.* **128**, 2161–2190 (2017).
13. D. Karlan, A. L. Ratan, J. Zinman, Savings by and for the Poor: A Research Review and Agenda. *Rev. Income Wealth.* **60**, 36–78 (2014).
14. D. Karlan, S. Mullainathan, B. N. Roth, Debt traps? Market vendors and moneylender debt in India and the Philippines. *Am. Econ. Rev. Insights* **1**, 27–42 (2019).
15. A. W. Cappelen, K. O. Moene, E. Ø. Sørensen, B. Tungodden, Needs versus entitlements—An international fairness experiment. *J. Eur. Econ. Assoc.* **11**, 574–598 (2013).

16. A. Barr, J. Burns, L. Miller, I. Shaw, Economic status and acknowledgement of earned entitlement. *J. Econ. Behav. Organ.* **118**, 40–54 (2015).
17. P. Jakiela, How fair shares compare: Experimental evidence from two cultures. *J. Econ. Behav. Organ.* **118**, 40–54 (2015).
18. J. P. Henrich, R. Boyd, S. Bowles, E. Fehr, C. Camerer, H. Gintis, *Foundations of Human Sociality: Economic Experiments and Ethnographic Evidence from Fifteen Small-scale Societies* (Oxford Univ. Press, 2004).
19. T. Yamagishi, N. Mifune, Y. Li, M. Shinada, H. Hashimoto, Y. Horita, A. Miura, K. Inukai, S. Tanida, T. Kiyonari, H. Takagishi, D. Simunovic, Is behavioral pro-sociality game-specific? Pro-social preference and expectations of pro-sociality. *Organ. Behav. Hum. Decis. Process.* **120**, 260–271 (2013).
